# Pharmacist-led antimicrobial stewardship programme in a small hospital without infectious diseases physicians

**DOI:** 10.1038/s41598-022-13246-6

**Published:** 2022-06-09

**Authors:** María Rosa Cantudo-Cuenca, Alberto Jiménez-Morales, Juan Enrique Martínez-de la Plata

**Affiliations:** 1 Pharmacy Department, Hospital Universitario Virgen de las Nieves, Av. de las Fuerzas Armadas, 2, 18014 Granada, Spain; 2 Pharmacy Department, Hospital de Poniente, El Ejido, Spain; 3 Department of Biochemistry and Molecular Biology II, University of Granada, Granada, Spain; 4 Pharmacy Doctoral Programme, University of Granada, Granada, Spain

**Keywords:** Antimicrobials, Infectious diseases

## Abstract

Pharmacists may be tasked to lead antibiotic stewardship programmes (ASP) implementation in small hospitals in absence of infectious diseases (ID) physicians. The objectives are to evaluate the effectiveness of a pharmacist-led ASP in a hospital without ID physician support, with special focus on indicators of the hospital use of antimicrobial agents based on consumption and asess the potential clinical and economic impact of pharmacist interventions (PIs) through the CLEO tool. A prospective quasi-experimental study to implement an ASP in a 194-bed hospital. We evaluated changes in antimicrobial use measured as mean defined daily doses per 1000 patient-days (AUD) for intervention versus preintervention period. A total of 847 antimicrobial PIs were proposed, being 88.3% accepted. Discontinuation due to excessive duration was the most frequently performed PI (23.4%). Most of PIs was classified as major or moderate clinical impact, 41.7% and 37.8% respectively. The global consumption of antimicrobial was reduced from 907.1 to 693.8 AUD, with a signifcant drop in carbapenems and quinolones. Direct expenditure of antibiotics decreased significantly. Pharmacist-led ASP has being effective in reducing consumption of antibiotics. In the absence of ID physician´s support and oversight, pharmacists could lead the improvement of the use of antimicrobials.

## Introduction

Antimicrobial resistance (AMR) is increasing worldwide which has become one of the most serious threats to public health. The overuse and misuse of antibiotics are associated with increased rates of adverse drug events, including *Clostridioides difficile* infection, and development of multidrug-resistant microorganisms, leading to longer hospital stays, greater mordibity and mortality and higher healthcare costs^1,2^. It is estimated that over 40-50% of prescriptions for antibiotics established in the hospital setting in and outside Europe are considered unnecessary, even when necessary, the antibiotics prescribed are often excessively broad spectrum or longer duration than necessary^3,4^. Taking into account that more than a half of patients may receive an antibiotic during their stay in hospitals^5^, it is necessary to implement specific actions that help optimise the use of antimicrobial.

Although AMR is not a new problem and in the last years governmental and regulatory agencies have developed strategies for and guidance on AMR^6,7^, the World Health Organization (WHO) has presented “Call to Action on Antimicrobial Resistance 2021” for enhanced global efforts and accelerate previous commitments to combat AMR.^8^ As reducing inappropriate antimicrobial utilisation has become a priority, most developed countries have implemented antibiotic stewardship programmes (ASP), that are coordinated interventions promoting the responsible use of antibiotics to improve patient outcomes and reduce antibiotic resistant bacterias^9^.

Guidelines published by the Infectious Diseases Society of America (IDSA) and the Society for Healthcare Epidemiology of America (SHEA), recommend a multidisciplinary team, including an infectious diseases (ID) physician, a hospital epidemiologist, a microbiologist and a clinical pharmacist with ID training^10^. Published reports about ASPs often pertain to programmes at large teaching hospitals, however few studies have examined the implementation theses programmes in smaller hospitals. One of the frontiers for implementing a formal ASP in these settings, where resources may be limited, is that oftentimes one or more of these professionals are not available^11^. In cases where there is not ID physician support, the responsibility for daily engagement in ASP activities is left to a pharmacist with ID training. In fact, multiple studies have indicated that the pharmacist can play a key role in promoting the optimal use of antimicrobial agents, monitoring and auditing the prescriptions, and educating health professionals^12,13^. A systematic review of ASPs with participation of clinical pharmacists implemented in small and medium-sized hospitals shows a significant decrease in the consumption and cost of antimicrobials^14^. Even if several studies have reported a notable increase of ID pharmacist participation in ASPs and an impact on optimising antimicrobial therapy after their interventions^14–16^, multiple barriers were identified in some countries^17^. Although the some studies have determined the impact of implementing a pharmacist-led ASP^12,18^, further research is needed of ASP driven by pharmacists.

Therefore, the objectives of this study are to evaluate the effectiveness of a pharmacist-led ASP in a small hospital without ID physician support, with special focus on indicators of the hospital use of antimicrobial agents based on consumption and asess the potential impact of pharmacist interventions (PIs) to improve antibiotic prescribing practices for hospital inpatients.

## Results

### Baseline characteristics

A total of 696 patients were included during the intervention period with a median age of 69.5 (IQR: 59–83) years and 41.2% of women, of whom 12.6% were allergic to antibiotics (68.2% of whom, to penicillins). A total of 847 antimicrobial PIs were proposed, of which pharmacist interacted directly with the prescriber in 5.4%. The median number of days from the start of treatment to the day of PI proposed was 4 (IQR: 1–8). Table 1 describes clinical and therapeutic characteristics of patients with PIs and physician acceptance rate. Patients were primarily admitted to the general internal medicine department (55.5%). Community-acquired infection was the most frequent acquisition type of infection (78.3%). Respiratory tract infection (RTI) (29.8%) was the most commonly focus of infection, followed by urinary tract infection (UTI) (25.7%) and biliary tract and intraabdominal infection (IAI) (17.2%). The therapy was empirical in 71.8% and polytherapy in 36.0%. Median treatment duration (prophylaxis not included) was 9 (IQR: 6-14) days. Sample collection was carried out in 66.8%. The intravenous administration route had the highest number of PIs (94.3%). Interventions associated with cephalosporins, penicillins, quinolones and carbapenems accounted for over one-third (67.8%) of all recommendations. The most common antimicrobials requiring modifications were levofloxacin (16.3%) and ceftriaxone (14.3%). Median duration of hospitalisation was eleven (IQR: 6-18) days. One third of patients (37.9%) were prescribed an antimicrobial agent at discharge, with a median duration of twenty (IQR:13-23) days and 32.8% of inappropiateness. Few patients (4.4%) were readmitted because of infection causes.

**Table 1:** Clinical and therapeutic characteristics of patients with pharmacist interventions.

Pharmacist interventions (n = 847)	n (%)	Acceptance (%)
**Hospital clinical departments**		
General internal medicine	470 (55.5)	403 (85.7)
General and Gastrointestinal Surgery	124 (14.6)	113 (91.1)
Urology	104 (12.3)	96 (92.3)
Traumatology and orthopedics	101 (11.9)	95 (94.1)
Intensive Care Unit	28 (3.3)	25 (89.3)
Others	20 (2.4)	16 (80.0)
Community-acquired infection	663 (78.3)	590 (89.0)
Healthcare-associated	184 (21.7)	158 (85.7)
**Clinical syndrome**		
Respiratory tract infection (RTI)	252 (29.8)	222 (88.1)
Urinary tract infection (UTI)	218 (25.7)	188 (86.2)
Biliary tract and intra-abdominal infection (IAI)	146 (17.2)	134 (91.7)
Osteoarticular infection (OAI)	83 (9.8)	81 (97.6)
Skin and soft tissue infection (SSTI)	42 (5.0)	32 (76.2)
Sepsis/Fever with no focus	29 (3.4)	23 (79.3)
Surgical site infection (SSI)	22 (2.6)	18 (81.8)
Gastrointestinal infection (GI)	9 (1.1)	7 (77.8)
Catheter-related bloodstream infection (CRBSI)	8 (0.9)	8 (100)
Other (e.g. central nervous system infection, infectious uveitis, etc.)	38 (1.5)	35 (92.1)
**Type of therapy**		
Empirical	608 (71.8)	536 (88.2)
Targeted	165 (19.5)	139 (84.2)
Prophylaxis	74 (8.7)	73 (98.6)
**Antimicrobial group**		
Cephalosporins	233 (27.5)	205 (88.0)
Quinolones	194 (22.9)	169 (87.1)
Penicillins	160 (18.9)	143 (89.4)
Carbapenems	87 (10.3)	70 (80.5)
Nitroimidazoles	54 (6.4)	52 (96.3)
Glycopeptides and lipopeptides	29 (3.4)	26 (89.7)
Lincosamides	26 (3.1)	25 (96.2)
Oxazolidinones	23 (2.7)	21 (91.3)
Antifungals	8 (0.9)	6 (75.0)
Aminoglycosides	7 (0.8)	6 (85.7)
Macrolides	6 (0.7)	6 (100)
Tetracyclines	6 (0.7)	6 (100)
Sulfonamides	6 (0.7)	5 (83.3)
Others	8 (0.9)	8 (100)

### Pharmacist antimicrobial interventions

The PIs classified by type are shown in Table 2. Discontinuation due to excessive duration was the most frequently performed PI (23.4%). The overall acceptance rate was 88.3%, 5.0% of PIs were rejected by physicians and 6.7% were not evaluable because of discharge or other reasons. Switching from intravenous to oral administration had the lowest acceptance rate (80.6%). Table 3 described the potencial impact of PIs through CLEO tool. Regarding the clinical impact, the number of avoids or fatality PIs was thirty (3.5%) e.g. daptomycin used to treat a complicated pneumonia, patient with a septic shock caused by *Pseudomonas* treated with ceftriaxone. Almost half were graded as major (41.7%) e.g., thrombocytopenia in a patient treated with linezolid, patient known to be allergic to beta-lactams treat with amoxicillin/clavunate. PIs classified as moderate were 37.8%, e.g. changing from intravenous to oral formulation, ciprofloxacin to treat an uncomplicated urinary tract infection. Minor or null significance PIs were 17.0%, e.g. discontinuation of metronidazole in combination with meropenem in a perforated appendicitis. No adverse events were noted after implementing a PI in any patient.

**Table 2:** Pharmacist interventions by intervention type and physician acceptance rate.

Pharmacist interventions (n = 847)	n (%)	Acceptance (%)
Discontinuation due to excessive duration	198 (23.4)	172 (86.9)
Therapy de-escalation	130 (15.3)	105 (80.8)
Dose adjustment or interval modification	128 (15.1)	128 (100)
Deleting an antibiotic of the complete treatment due to use of redundant antimicrobial therapy	103 (12.2)	97 (94.2)
Switching from intravenous to oral administration	93 (11.0)	75 (80.6)
Changing the empirical therapy because of inappropriateness	85 (10.0)	72 (84.7)
Therapeutic escalation	58 (6.9)	55 (94.8)
Discontinuation due to a lack of indication to proceed	44 (5.2)	37 (84.1)
Others	8 (0.9)	7 (87.5)

**Table 3:** Potencial clinical, economic and organisational impact of pharmacist interventions through CLEO tool.

	n (%)
**Clinical impact**	
Negative	0 (0)
Null	49 (5.8)
Minor	95 (11.2)
Moderate	320 (37.8)
Major	353 (41.7)
Avoids / Fatality	30 (3.5)
**Economic impact**	
Increase in cost	153 (18.1)
No change	23 (2.7)
Decrease in cost	671 (79.2)
**Organisational impact**	
Negative	128 (15.1)
Null	317 (37.4)
Positive	402 (47.5)

### Clinical and economic outcomes of antimicrobial stewardship programmes

Changes in antimicrobial use and expenditure are described in Table 4. The global consumption of antimicrobials was signifcantly reduced from 907.1 AUD in the pre-intervention period to 693.8 AUD in the intervention period (-23.5%), with a signifcant drop in carbapenems (73.3 vs 34.9 AUD; p=0.012) and fluoroquinolones (181.9 vs. 95.8 AUD; p=0.012). Overall consumption of antibacterial agents was reduced by 23.1% (874.6 vs. 672.5 AUD; p=0.012). The ratio anti-Methicillin-sensible *Staphylococcus aureus* (MSSA) agents (cloxacilin + cefazolin) / anti*-*MRSA agents (glycopeptides, daptomycin, linezolid, tedizolid, dalbavancin and ceftaroline) was increased (1.3 vs 1.8; p=0.025). No differences in antimicrobial resistance trends and *Cl.difficile* were found. Direct expenditure of antibiotics decreased significantly (p=0.012). The total saving between pre-intervention period (616356 €) and intervention period (451402 €) was 164953 €.

**Table 4:** Indicators of the hospital use of antimicrobial agents based on consumption and expenditure in the pre-intervention and intervention periods.

AUD, mean/trimester (SD)	Preintervention^a^	Intervention^b^	*p*-value
Overall consumption of antimicrobials	907.1	693.8	0.012
Overall consumption of antibacterial agents	874.6	672.5	0.012
Overall consumption of systemic antifungal agents	32.5	21.3	0.069
Consumption of carbapenems	73.3	34.9	0.012
Consumption of fluoroquinolones	181.9	95.8	0.012
Consumption of macrolides	31.1	35.9	0.401
Consumption of metronidazole	32.1	19.6	0.069
Consumption of phosphomycin	1.9	5.4	0.012
Sequential therapy	0.4	0.5	0.484
Anti-MSSA agents/anti-MRSA agents ratio	1.3	1.8	0.025
Amoxicillin/amoxicillin-clavulanic acid ratio	0.1	0.1	0.779
Amoxicillin-clavulanic acid/piperacillin-tazobactam ratio	4.6	3.7	0.093
Macrolides/fluoroquinolones ratio	0.2	0.5	0.025
Direct acquisition cost (€), mean/trimester (IQR)	77044.5	56425.3	0.012

^a^January 1, 2017 to December 31, 2018.

^b^January 1, 2019 to December 31, 2020.

*AUD* Defined daily doses (DDD) per 1000 patients-days. *IQR* Interquartile range, *MRSA* Methicillin-resistant *Staphylococcus aureus*, *MSSA* Methicillin-susceptible *Staphylococcus aureus*, *SD* Standard deviation.

## Discussion

In this study, we have showed the impact of a pharmacist-lead ASP, demonstrating that it can be effective in a small hospital, where the shortage of ID physicians is a major impediment to implement an ASP. Although the programmes involving clinical pharmacists in hospitals have been effective in decreasing both antimicrobial use and the cost^14,19^, little evidence have shown the efectiveness of an ASP led by pharmacists without ID physician support. A study involving only a full-time ID pharmacist has indicated substantial decreases in the utilisation of carbapenems, daptomycin, echinocandins and levofloxacin, in addition to cost savings^12^. In this study^12^, the acceptance rate (91.8%) have been slightly higher than the observed in our study (88.3%),in accordance with the acceptance level usually reported (from 70.0 to 97.5%)^20^. Significant reductions in the use of special-vigilance drugs, such ascarbapenems and linezolid, and overall antimicrobial cost have been reported in other study that analyse the PIs to improve appropriate antibiotic prescribing in a 164-bed hospital (acceptance rate: 83.4%)^14^. There has been a significant drop in AUD of carbapenemic agents during the intervention period. This fact is an important achievement, since this class of antibiotics is considered of last resort. Moreover, we have reached a significant reduction in overall consumption of antimicrobials, including carbapenems, fluoroquinolones and anti-MRSA agents, similarly to other studies^21^. Besides, during the postimplementation period, the relative reduction in antimicrobial expenditures was approximately 165000 €, that is a decrease of 26.8%.

Unlike other studies that drug dose adjustment was the most common PI^22,23^, we have proposed the discontinuation of therapy due to excessive duration in almost quarter of interventions, being the most frequent PI. Nowadays, there is an evidence-based dogma of “shorter is better”, as short-courses of antimicrobial therapy have been shown to be equivalent in efficacy to longer therapies, reducing selective pressures of antibiotics^24^, A recent systematic review has analysed the effectiveness of pharmacist-led interventions aimed at improving antimicrobial usein hospital patients, showing greatest guidelines compliance and reducing duration of antimicrobial therapy^25^, Given that there is a strong evidence for the reduction of the duration of antibiotic treatments for community-acquired pneumonia and acute exacerbation chronic bronchitis, respiratory tract infections have been the most frequent clinical syndrome in which pharmacists have proposed PIs, and cephalosporins, the main drug class. On the other hand, fluoroquinolones account for almost a quarter of antimicrobial PIs (22.9%), being the antibiotic class with the largest AUD reduction. Although ciprofloxacin is one of the most effective antibiotic to treat urinary tract infections (UTIs), the prevalence of fluoroquinolone resistance among *Escherichia coli* and *Klebsiella* spp. in our area issignificantly high.

## Limitations

Our study has several limitations. First, this is a single-center observational study, so generalisability to other small and medium-sized hospitals must be taken with caution Second, we have not evaluated clinical outcomes, such as length of stay, readmission rates and mortality. However, most studies of ASP have limited association with clinical outcomes and it may be difficult to relate causally to specific ASP activities, being able to be influenced by multiple factors^12,25^. Third, a longer follow-up period is generally required to observe changes in antimicrobial resistance. Besides, it can also be influenced by the presence of confounding variables such as other control measures of infections. Despite the potential limitations previously mentioned and although the conclusions of this study are limited by the quasiexperimental design, our results could suggest an association between the ASP activities and the significant improvement of the indicators of the antimicrobial agents hospital use.. Our study is the first in which these indicators proposed by a panel of experts, through a Delphi method combined with scientific evidence, have been applied in the hospital setting. Although the identification of quality indicators with enough applicability and reliability is still one of the developing areas in antimicrobial stewarship and the use of these indicators should be made widespread, data provided can be useful to implement actions of improvement and evaluate the impact of antibiotic policies.

## Conclusion

The findings of this study highlight the relevance of ID pharmacists in ASP. Pharmacist-led ASP has achieved a reduction in the overall consumption of antimicrobials, specially carbapenems and fluoroquinolones, as well as, other key indicators. PIs carried out to improve the use of antimicrobials positively impact on clinical and economic outcomes, with a high acceptance by physicians. Therefore, in the absence of ID physician´s support and oversight, pharmacists could be key in the improvement of the use of antimicrobials.

## Method

### Study design and participants

**Figure 1:**
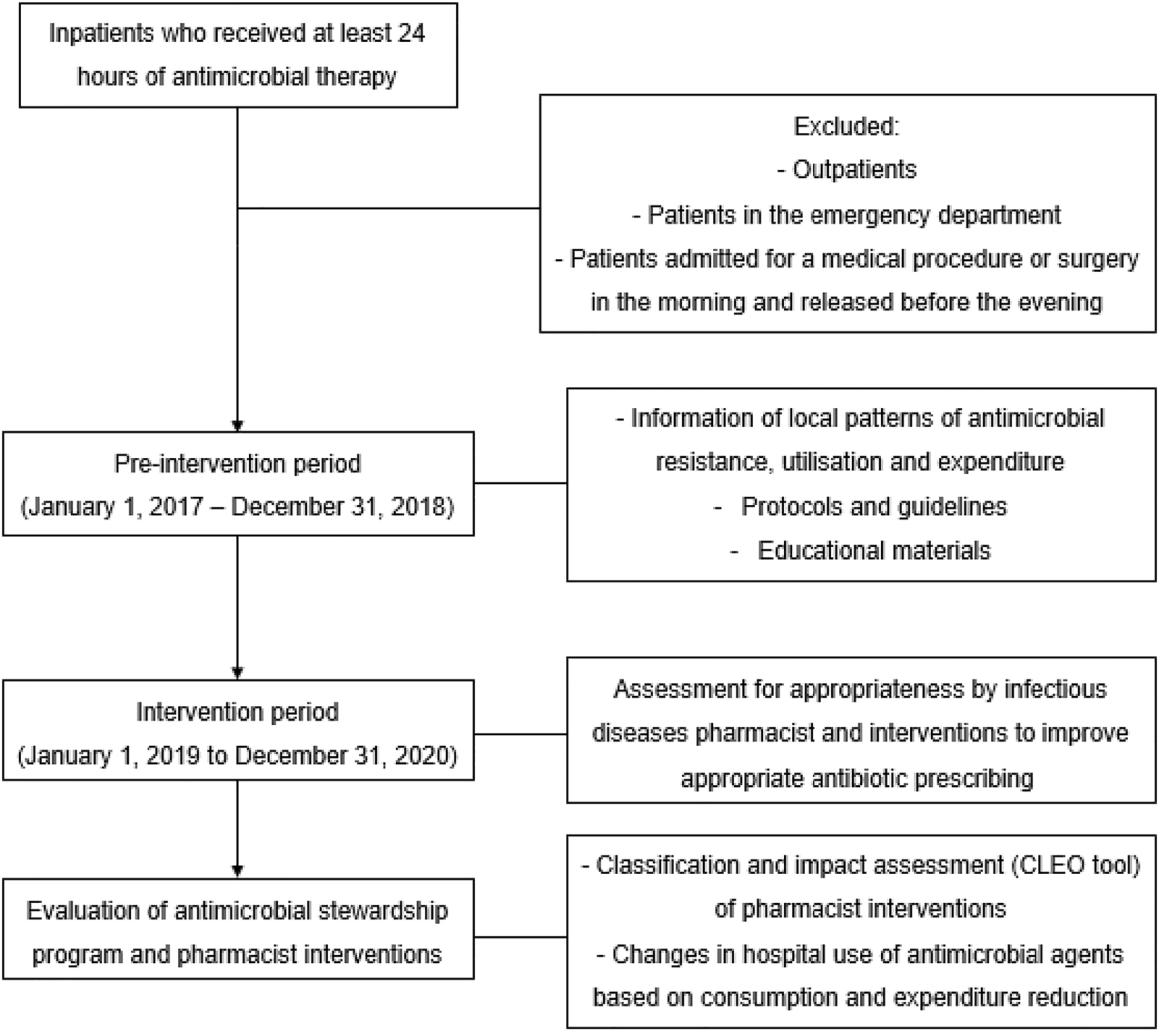
Study design.

A prospective quasi-experimental study was conducted to implement an ASP in a public 194-bed hospital in Spain. The ASP was driven by an ID trained clinical pharmacist in collaboration with a preventive medicine physician and a microbiologist, but without an ID physician. All inpatients who received at least 24 hours of antimicrobial therapy were included. Study design is represented in Fig. 1. We excluded outpatients, patients in the emergency department and those admitted for a medical procedure or surgery in the morning and released before the evening. For PIs analysis, any discharged patient who was readmitted during the study period was considered as a new patient.

The study period was divided into two periods of 24 months each. During the pre-intervention period (January 1, 2017–December 31, 2018), baseline information of local patterns of antibiotic resistance, antimicrobial utilisation and expenditure were collected. ASP team revised prophylaxis and antibiotic therapy in management protocols and developed guidelines with local antimicrobial recommendations. Second phase (intervention period), aimed at improving appropriate antibiotic use, took place from January 1, 2019 to December 31, 2020. Sessions were held between the ID pharmacist and professionals of each hospital service to present the ASP and the guidelines, just to provide educational materials (i.e. leaflet on hand hygiene, booklets for antibiotic prescribing) and antimicrobial consumption and resistance data. Furthermore, during the intervention period, every day (Monday-Friday) the ID pharmacist identified hospitalised patients with prescriptions of drugs belonging to Anatomical Therapeutical Chemical Classification System (ATC) class J01 (antibacterials for systemic use) and class J02 (antimycotics for systemic use), through the electronic prescribing,to optimise antimicrobial use.

### Data collection

A digital report was exported daily including information on admission date, admitting hospital department and antimicrobial agent prescribed with dosage, route and frequency. From electronic medical records, we also obtained demographic and clinical characteristics of patients including sex, age, allergic reactions to antibiotics, as well as, variables related to infection and antimicrobial prescription such as infectious diagnosis on admission, healthcare-associated or community-acquired infection, type of therapy (empirical, targeted or prophylaxis), treatment duration, and laboratory and microbiological data. Patients were followed up until discharge and we recorded duration of hospitalisation and antimicrobial prescribed at discharge (class, duration and appropriateness).

#### Pharmacist antimicrobial interventions

Each antimicrobial prescription was assessed for appropriateness by ID pharmacist according to the guidelines. Then, the pharmacist performed and recorded PIs in the electronic prescribing, focused on highly restricted drugs like carbapenems, anti-Methicillin-resistant *Staphylococcus aureus* (MRSA) and fluoroquinolones^26^, and prescriptions for more than 10 days. When necessary, the pharmacist interacted directly with the prescriber in person or by phone. The proposed PIs and the physician’s acceptance or rejection were categorised into the following types:

1. Dose adjustment or interval modification, including renal and/or hepatic disease adjustments and pharmacokinetic/ pharmacodynamic reasons.
2. Switching from intravenous to oral administration.
3. Changing the empirical therapy because of inappropriateness (i.e. substitution with a more appropriate antimicrobial to optimise efficacy or toxicity, change to equivalent most cost-effective regimen).
4. Therapeutic de-escalation (switching the drug to an antibiotic with narrower antimicrobial activity spectrum upon identifying the infecting pathogen).
5. Therapeutic escalation (switching the drug to a broad spectrum antibiotic because the pathogens identified are resistant to administered treatment).
6. Discontinuation due to excessive duration (days of therapy beyond the indicated duration of therapy without any clinical reason for a lengthened course)
7. Discontinuation because a lack of indication to proceed (i.e. use of antimicrobials for non-infectious syndromes or or antibiotics for non-bacterial infections, palliative situation).
8. Deleting an antibiotic of the complete treatment due to use of redundant antimicrobial therapy.
9. Other interventions: therapeutic drug monitoring, interactions, allergies, etc.

To assess the potential impact of PIs, we utilised the CLEO tool^27^, a comprehensive tool assessing clinical, economic and organisational impact of PIs which has been developed, validated and was reliable and feasible for use in routine clinical practice.

#### Clinical and economic outcomes of antimicrobial stewardship programmes

To evaluate changes in hospital use of antimicrobial agents based on consumption^28^ for intervention versus preintervention period, we used mean defined daily dose (DDD) per 1000 patient-days (AUD), calculated as follows: [Total dose (grams) of antimicrobial used / DDD x Total days of hospital stay] x 1000. DDDs were calculated using World Health Organization (WHO) definitions. We also analysed the expenditure reduction for intervention versus preintervention period calculated according to hospital’s acquisition direct cost, as well as, antimicrobial resistance (MRSA, extended spectrum beta-lactamase phenotypes (ESBL) in *Escherichia coli* and *Klebsiella* *pneumoniae*, Carbapenemase-producing Enterobacteriaceae, multidrug-resistant *Pseudomonas aeruginosa*) and *Cl.difficile*.

### Statistical analysis

Descriptive statistics were used to evaluate the characteristics of the sample. Qualitative variables are expressed as relative and absolute frequency distributions. Continuous variables are expressed as mean (standard deviation; SD) or median (interquartile range; IQR) in case of asymmetry. The χ2 test or Fisher’s exact test, as appropriate, was used to compare categorical data and the Student's t-test for normally distributed continuous variables and Mann-Whitney U test for non-normally distributed continuous variables. Statistical significance was considered if p values were less than 0.05. For the statistical analysis, the software SPSS Statistics for Windows, Version 21.0 (IBM Corp, Armonk, NY, USA) was used (https://www.ibm.com/support/pages/spss-statistics-210-available-download).

### Ethics approval

The study was conducted according to the guidelines of the Declaration of Helsinki, and approved by the Ethics Committee of Jaén Province. Informed consent was obtained from all subjects involved in the study or their legal representatives.
